# Correspondence

**Published:** 1883-03

**Authors:** 


### CORRESPONDENCE.

EDITOR REGISTER.—I notice an article in the REGISTER for January, in which the writer places considerable stress on the construction of the flask, to secure "good joints in block teeth work." In our opinion, any good flask, Hays' for instance, is all that is necessary, so far as the flask is concerned. When the teeth are properly jointed, and good plaster is used, with sufficient "vents" for the escape of surplus rubber, any flask will carry the piece safely through, and that too, without the necessity of an "examination during the process of closing the flask," provided it is properly warmed for the reception of the base.

I have noticed in recent issues of the REGISTER, several different "plans" by which a set of teeth *might* be antagonized; please permit me to give my experience in this work, for the failure to antagonize properly—this is the "rock" upon which a great many otherwise *good* pieces of plate work "split." There should be no doubt of the correctness of the "articulation" on the articulators, which, if correctly made, is certain to require no "grinding" when the work is finished, and no pains or work should be spared to accomplish this end.

For a full set, place rim of wax around the rim of each model, wax down well to trial plate—place upper plate in mouth and add or trim off wax to the length of upper lip—take out and insert under plate and trim down until just on level with under lip—then with both plates in mouth, trim down or build up until they come together with *some* pressure *all* around—add or trim off to restore the contour--throw the head far back, and direct to open and close the mouth, (don't say naturally,) draw a line across each rim, open and close again and again, until satisfied the closure is the same each time—mark the center and sides, take out and place together, *as in the mouth;* place on the articulators, leaving small space between the top model and rim of the frame, and be sure there is no "screw loose;" cut out blocks of wax and insert blocks of teeth, alterately in each section, remembering that the fulness and length of wax represents fullness and length of teeth. As each block is placed in position, press rims of wax (and teeth already on) *firmly* together, and when waxed up, the two sections will close on pressure, and separate when it is removed.

In trimming for "vents," bevel the investing plaster from the edge of plate, back to rim of flask, in both sections, all around, cutting out plentifully toward the rim, leaving the space between the sections, *near the plate*, thin enough for the surplus material to break off easily when the piece is vulcanized; in this way, the surplus has free "vent" and can be disposed of as it comes from the plaster, without the use of the knife, saw, or file, and when placed in the mouth, there will be "not a little or no grinding," but *no* "grinding," and perfect joints. P. H. CALLAHAN,

Guling, Texas.

---

EDITOR REGISTER--*Dear Sir*:--Would you advise the extraction of a temporary cuspid of a child thirteen years of age, where it is firm in position, the permanent not having as yet made any appearance, except a slight protuberance above the temporary tooth? On the opposite side the permanent tooth is well in place.

Yours truly, J. P. C.

In such a case as that described above, the temporary tooth should not be extracted till the permanent tooth gives some indication of its approach, by causing prominence of the gum, or producing loosening of the temporary. Until the permanent tooth gives some indication of the fact, it can not be known whether there is a tooth, or a germ even, in the jaw at this point; and even though the tooth is present it may be in such a position that it could never occupy properly the place for which it was designed. A temporary cuspid that will be supported and remain firm is far better than a vacancy.

---

EDITOR REGISTER:—A peculiar case of abrasion of the enamel lately came under my notice. The patient was a lady, aged forty, with the two superior central incisors roughened; with the nail a slight depression could be felt, and the polished appearance of the enamel was gone. This extended over about one-third of the labial surface of the teeth next the gum. No other teeth were involved. The lady is a person of good health, regular habits, good digestion, etc. Teeth of the dense, yellowish class, rather better than the average.

On inquiry, could not find that any acid fruits, other than grapes, had been eaten; or any medicines to injure the teeth, been taken, as homœopathic treatment is followed in the family (!)

Saliva was normal, both in quantity and quality. Found nothing harmful in a dentrifice she had been using. After polishing well with prepared chalk and a wood point, followed by a hard rubber cone, the patient was dismissed. Was seen again in three or four days after, when the roughened appearance had somewhat disappeared, though not entirely gone.

Have any of the readers of the REGISTER seen a similar case? and can they tell what is the agent which caused it? and the probability of its recurrence? K. C. M.

